# Predictors of Recurrence and Patterns of Initial Failure in Localized Ewing Sarcoma: A Contemporary 20-Year Experience

**DOI:** 10.1155/2021/6681741

**Published:** 2021-04-17

**Authors:** Gregory C. Stachelek, John A. Ligon, Jennifer Vogel, Adam S. Levin, Nicolas J. Llosa, Brian H. Ladle, Christian F. Meyer, Stephanie A. Terezakis, Carol D. Morris, Matthew M. Ladra, Christine A. Pratilas

**Affiliations:** ^1^Department of Radiation Oncology and Molecular Radiation Sciences, Johns Hopkins University School of Medicine, Baltimore, MD, USA; ^2^Department of Pediatric Oncology, Johns Hopkins University School of Medicine, Baltimore, MD, USA; ^3^Department of Orthopedic Surgery, Johns Hopkins University School of Medicine, Baltimore, MD, USA; ^4^Department of Medical Oncology, Johns Hopkins University School of Medicine, Baltimore, MD, USA

## Abstract

**Background:**

The majority of patients with localized Ewing sarcoma will remain disease-free long term, but for those who suffer recurrence, successful treatment remains a challenge. Identification of clinicopathologic factors predictive of recurrence could suggest areas for treatment optimization. We sought to describe our experience regarding predictors of recurrence and patterns of first failure in patients receiving modern systemic therapy for nonmetastatic Ewing sarcoma.

**Methods:**

The medical records of pediatric and adult patients treated for localized Ewing sarcoma between 1999 and 2019 at Johns Hopkins Hospital were retrospectively analyzed. Local control was surgery, radiotherapy, or both. Recurrence-free survival (RFS) was calculated using the Kaplan–Meier method. Univariable and multivariable Cox proportional-hazards modeling was performed to obtain hazard ratios (HR) for recurrence.

**Results:**

In 94 patients with initially localized disease, there were 21 recurrences: 4 local, 14 distant, and 3 combined. 5-year and 10-year RFS were 75.6% and 70.5%, respectively. On multivariable analysis including age at diagnosis and tumor size, <95% tumor necrosis following neoadjuvant chemotherapy (NAC; HR 14.3, *p* = 0.028) and radiological tumor size change during NAC (HR 1.04 per 1% decrease in size change, *p* = 0.032) were independent predictors of recurrence. Among patients experiencing distant recurrence, pulmonary metastases were present in 82% and were the only identifiable site of disease in 53%.

**Conclusions:**

Poor pathologic or radiologic response to NAC is predictive of recurrence in patients with localized Ewing sarcoma. Suboptimal tumor size reduction following chemotherapy provides a means to risk-stratify patients who do not undergo definitive resection. Isolated pulmonary recurrence was a common event.

## 1. Introduction

Ewing sarcoma (ES), the second most common primary tumor of bone in children and adolescents, is a rare, aggressive neuroectodermal malignancy with a high propensity for metastasis to lungs, bone, and bone marrow [1, 2]. Approximately 25% of patients with ES have overt metastases at diagnosis, while the remainder have clinically localized disease [3]. Though patients who present with metastatic disease have dismal outcomes [4], systemic therapy intensification has profoundly improved cure rates for individuals with localized ES. 5-year overall survival in children and adolescents with ES now reaches 70–75% [5–7] and is only slightly lower in the adult population [8].

In the United States, multiagent chemotherapy paired with appropriate local control is well-established as the current treatment paradigm for localized ES in children and adolescents [5, 6, 9]. In the regimen tested in the Children's Oncology Group AEWS0031 trial of interval compression, considered standard by many U.S. sites, six two-week cycles of interval compressed neoadjuvant chemotherapy (vincristine, doxorubicin, and cyclophosphamide alternating with ifosfamide and etoposide [VDC-IE]) are followed by definitive local control (surgical resection, radiotherapy [RT], or surgery followed by radiation) and eight additional cycles of consolidation VDC-IE. Similar regimens have been employed in adult ES patients [10].

While local recurrence rates are low with definitive management [11, 12], distant treatment failure remains a clinical challenge and affects up to 30–40% of patients with initially localized disease [10]. Salvage therapies are often ineffective and survival following recurrence remains poor [13, 14]. Patients over the age of 18 years, those with a primary pelvic tumor, those with larger tumors, and those not treated with ifosfamide/etoposide have been identified as at particularly high risk for relapse [15, 16]; however many such retrospective series included patients who were treated prior to contemporary ES treatment paradigms.

Histologic response to neoadjuvant chemotherapy (NAC) has been cited as a predictive factor in localized ES [17]. Several studies have associated inferior extent of tumor necrosis following NAC with recurrence risk in localized ES [18–20]. The quantitative threshold defining optimal histologic necrosis has not been employed uniformly across studies, however. Further, pathologic assessment of tumor necrosis is limited to patients who underwent surgery and whose tumors may therefore be examined *ex viv*o. Because definitive RT is considered an acceptable method for local control in select patients [21–23], other readily assessable clinical factors predictive of recurrence may be of high utility [24, 25].

We therefore performed a retrospective review of patients with Ewing sarcoma treated at the Johns Hopkins Hospital (JHH), in order to identify factors associated with recurrence of localized ES in a contemporary cohort of pediatric and adult patients treated with modern systemic therapy regimens and any local control modality. We sought to characterize sites of first recurrence in patients with distant failure. Improved risk stratification of patients with nonmetastatic ES, irrespective of surgical status, may suggest potential for risk-directed therapeutic escalation.

## 2. Materials and Methods

### 2.1. Study Population

We retrospectively reviewed the medical records of patients who underwent treatment for Ewing sarcoma at Johns Hopkins Hospital (JHH). Study inclusion criteria were pathologic confirmation of Ewing sarcoma or Ewing sarcoma-family tumor (ESFT), no clinical or radiologic evidence of metastatic disease, and first oncology consultation between January 1, 1999, and June 30, 2019. Patients fitting the inclusion criteria were identified using databases of the pediatric oncology, medical oncology, and radiation oncology treatment teams as well as through diagnostic coding of “Ewing sarcoma” in the electronic medical record. All retrospective data collection was approved by the JHH Institutional Review Board (IRB #00227515).

Clinical data collection included year of diagnosis, patient age at diagnosis, patient sex, EWSR1 translocation status, anatomic site and size of primary tumor at diagnosis, erythrocyte sedimentation rate (ESR) and lactate dehydrogenase (LDH) serum levels at diagnosis, definitive local therapy modality, percent change in primary tumor size following NAC, percent necrosis of resected tumor specimens following NAC, and date of first recurrence, where applicable.

### 2.2. Radiology

All patients underwent dedicated computed tomography (CT) and/or magnetic resonance imaging (MRI) for delineation of the primary tumor extent prior to treatment, as well as following neoadjuvant chemotherapy but prior to definitive surgery or RT. Metastatic staging was performed at the same time points and included, at a minimum, CT imaging of the chest. Whole-body technicium-99 bone scan or fluorodeoxyglucose positron emission tomography (FDG-PET) scan was also available in a subset of patients, but those lacking whole-body imaging modalities were not excluded from analysis. Patients with evidence of metastatic disease on any imaging modality at the time of presentation were omitted from the analysis. Assessment of radiological response following NAC was based on shrinkage of the largest tumor dimension at time of presentation, though the dimension in question was held consistent (i.e., if the tumor was largest along the craniocaudal axis, this was also the axis that was remeasured following NAC, even if it no longer remained the largest tumor dimension).

### 2.3. Chemotherapy

The majority of patients (93%) received systemic therapy regimens that included vincristine, doxorubicin, and cyclophosphamide alternating with ifosfamide and etoposide (VDC-IE). Up to six cycles of VDC-IE were delivered prior to definitive surgery or RT. Dosage and frequency generally reflected the currently accepted standard of care per contemporary Children's Oncology Group protocols.

### 2.4. Radiation Therapy

Patients treated with definitive RT to the primary tumor underwent either 3D conformal RT or intensity-modulated RT to a median dose of 55.8 Gy in 31 fractions (range 52.2–57.6 Gy). Whole lung irradiation (WLI) was delivered to a median dose of 15 Gy in 10 fractions (range 15–18 Gy) using opposed anterior-posterior fields covering the entirety of the unilateral or bilateral hemithoraces.

### 2.5. Statistical Analysis

Summary statistics were calculated for all patients. Survival analyses were conducted for recurrence-free survival (RFS), event-free survival (EFS), and overall survival (OS) using the Kaplan–Meier method and, when stratified by clinical characteristics, differences were compared using the log-rank test. Events of interest for RFS included mode of first failure (local, distant, or both) and anatomic site of first failure, and events of interest for EFS included any local or distant recurrence, development of second malignancy, or death. Univariable Cox regression analysis was conducted to identify clinical variables associated with RFS. Odds ratios for radiographic size change were defined relative to 100% decrease in radiographic size (i.e., no residual tumor on imaging), with decrease in size change therefore reflecting less tumor shrinkage. Variables with higher association on univariable analysis were included in multivariable models, allowing for approximately one variable per 10 events. Local and distant recurrence were assessed radiologically by growth of an existing lesion or appearance of new lesions following definitive therapy. All statistical analyses were conducted using *R* (version 3.6.2). *p* values less than 0.05 were considered statistically significant.

## 3. Results

### 3.1. Patient and Treatment Characteristics

**Figure 1:**
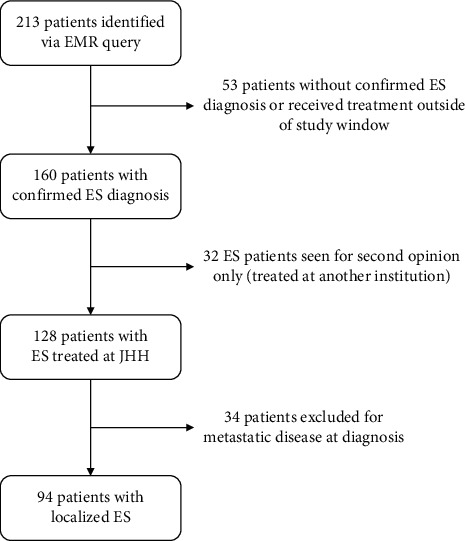
Flowchart of identification of patients with localized Ewing sarcoma (ES) treated at Johns Hopkins Hospital (JHH). EMR: electronic medical record.

A total of 94 patients with localized ES who met study inclusion criteria were identified. The patient identification process is detailed in Figure 1. Patient and treatment characteristics are summarized in Table 1. 51% of patients were male and the median age at ES diagnosis was 18.0 years (range 0.1–70.4 years). 81% of patients underwent surgical resection (with or without RT) for local control.

### 3.2. Clinical Outcomes

**Figure 2:**
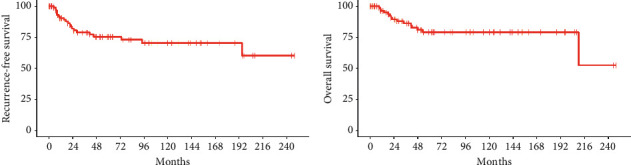
Kaplan–Meier curves of (a) recurrence-free survival and (b) overall survival for the entire cohort of 94 patients with localized Ewing sarcoma.

Median follow-up was 44 months for the entire cohort and median follow-up for living patients was 53 months. Twenty-one (22%) patients experienced a recurrence over the study period. Four patients experienced isolated local recurrence, 14 experienced isolated distant recurrence, and three had combined local and distant recurrences. Kaplan–Meier plots of RFS and OS for the entire cohort are shown in Figure 2. RFS and OS were 75.6% and 79.0% at 5 years and 70.5% and 79.0% at 10 years, respectively.

There were 12 deaths among patients who experienced recurrence, all attributable to disease progression. There were three deaths among patients who remained recurrence-free, two attributable to development of a second malignant neoplasm (SMN) and one of unidentified cause in a patient with no evidence of residual or recurrent ES. There were six SMNs: two osteosarcomas, three acute myeloid leukemias, and one synovial sarcoma.

### 3.3. Risk Factors for Recurrence

**Figure 3:**
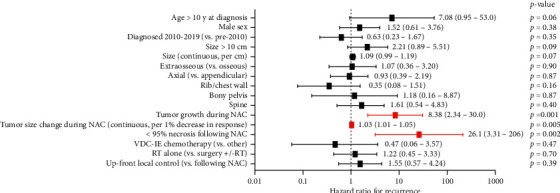
Forest plot of putative risk factors for recurrence of localized Ewing sarcoma. Odds ratios for recurrence with 95% confidence intervals are shown and were obtained by univariable Cox regression modeling. NAC: neoadjuvant chemotherapy; VDC-IE: vincristine, doxorubicin, cyclophosphamide, ifosfamide, etoposide; RT: radiotherapy.

Univariable Cox regression analysis was performed to identify clinical, radiologic, and pathologic variables associated with ES recurrence (Figure 3). Tumor growth on imaging following NAC (OR 8.38, *p*=0.001), percent change in maximal tumor dimension following NAC (OR 1.03 per 1% decrease, *p*=0.005), and <95% tumor necrosis on surgical pathology following NAC (OR 26.1, *p*=0.002) were each a significant predictor of recurrence on univariable analysis.

Change in tumor size and <95% tumor necrosis in pathologic specimens remained significant predictors on multivariable Cox regression analysis incorporating age at diagnosis and initial tumor size (OR 1.04 per 1% decrease, *p*=0.032 and OR 14.3, *p*=0.028, respectively). There was a strong positive correlation between increasing degree of radiographic response and good (95–100% necrosis) pathologic response on Pearson analysis (*r* = 0.435, *p*=0.005).

**Figure 4:**
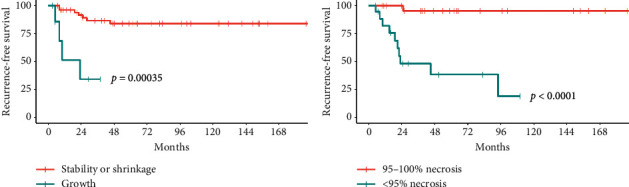
Kaplan–Meier curves of recurrence-free survival in patients with localized Ewing sarcoma stratified by (a) tumor growth vs. tumor stability or shrinkage following NAC and (b) ≥95% necrosis vs. <95% necrosis following NAC.

Kaplan–Meier plots of RFS stratified by tumor growth during NAC vs. no growth or shrinkage (*p*=0.00035 by log-rank test) and by 95–100% tumor necrosis vs. <95% tumor necrosis (*p* < 0.0001 by log-rank test) are shown in Figure 4.

Larger initial tumor size at diagnosis had a trend towards inferior outcome but did not reach significance (OR 1.09 per 1 cm increase, *p*=0.07). Age at diagnosis, patient sex, anatomic site of primary tumor, and receipt of RT as definitive therapy (versus surgery ± RT) also failed to demonstrate a significant association with local or distant recurrence in our cohort.

### 3.4. Patterns of Failure

Twenty-one patients in our localized ES cohort experienced disease recurrence, including 17 patients with distant failure (three with concomitant local recurrence). The lungs were the most common site of distant recurrence, with pulmonary metastases noted in 14 patients (82%) at time of failure (Table 2). Nine patients (53%) had no evidence of local or distant disease aside from pulmonary metastases. Bone was the next most common site of initial distant failure, occurring in five patients (29%). 5-year post-recurrence survival in patients with initially localized ES was 25%.

We further compared these patterns of failure to the sites of first progression or recurrence in 34 additional patients with ES who presented with metastases at the time of initial diagnosis. Twenty patients (59%) developed progression or recurrence following treatment, of whom 19 had distant metastases at the time of first failure. The lungs were again the most common site of distant failure, in nine patients (47%), with four patients (21%) with pulmonary-only recurrence.

### 3.5. Whole Lung Irradiation for Pulmonary Metastases

Four patients in our cohort with localized ES received salvage whole lung irradiation (WLI) following pulmonary recurrence: two (50%) had clearance of pulmonary disease and remained alive and disease-free at 118 and 138 months after recurrence. Eleven patients with initially metastatic disease involving the unilateral or bilateral lungs underwent WLI as part of their definitive therapy: eight (73%) had clearance of pulmonary disease and seven (64%) remained alive.

## 4. Discussion

The identification of patients with localized Ewing sarcoma who are most likely to experience recurrence remains a clinical challenge. Here, we describe our institutional experience regarding predictors of disease recurrence and patterns of failure in patients with localized ES. Our work demonstrates that poor response to neoadjuvant chemotherapy, as assessed by either lack of radiographic tumor shrinkage or less than 95% tumor necrosis on surgical pathology specimens, is highly associated with recurrence of ES. We further show that a large majority of patients who experience distant recurrence first present with pulmonary metastases, many of whom have no other radiographic evidence of disease.

The association of incomplete response to chemotherapy with inferior outcomes has been recognized in several prior ES cohorts. In particular, increasing degree of tumor necrosis on pathologic specimens correlates with decreased local and distant recurrence of initially localized ES. Varied cutoffs have been utilized to define poor response, however, including anything less than complete (100%) tumor necrosis [20]. Our work reinforces these findings and demonstrates that ES patients with 95% or greater necrosis on resection specimens have significantly improved recurrence-free survival as compared to those for whom less than 95% necrosis is observed. Whether a strong pathologic response is predictive of improved RFS even in patients with poor radiographic response is an intriguing question. Owing to the very low number of recurrences in patients with 95–100% tumor necrosis following NAC, we are not able to draw any firm conclusions regarding the superiority of pathologic response over radiographic response in predicting treatment success in such patients. This question would be interesting to examine in a larger multi-institution ES cohort.

The use of radiologic assessment to gauge response to systemic therapy and predict recurrence in ES patients remains controversial. While some Ewing sarcomas may regress dramatically in response to neoadjuvant chemotherapy, lack of shrinkage does not always correlate with therapeutic efficacy as assessed by necrosis. We show, however, that a simple radiographic measure of decreased tumor size (percent decrease in maximal tumor dimension between prechemotherapy CT or MRI and follow-up imaging immediately prior to definitive local therapy) is associated with decreased risk of recurrence. This measure may offer the opportunity to better risk-stratify those patients who are not surgical candidates based on tumor location or medical comorbidities.

Over 80% patients in our study who experienced distant recurrence of initially localized disease (with or without concomitant local failure) presented with pulmonary metastases as a site of first failure. While this figure is striking, multiple studies and clinical trials have also shown a preponderance of pulmonary failures [26–30]. Importantly, 53% of patients who suffered from distant failure had no identifiable extrapulmonary disease. Relatively few patients with pulmonary recurrence or pulmonary metastases at diagnosis underwent whole lung irradiation; however, those who did had favorable rates of durable pulmonary clearance.

Interestingly, we did not observe any statistically significant association between risk of recurrence and either patient age at the time of diagnosis or the anatomic location of the primary tumor. Patients with a primary tumor in the pelvis were significantly more likely to present with metastatic than localized disease, but among patients with localized ES, there was no difference in risk of recurrence between axial vs. appendicular, osseous vs. extraosseous, rib vs. nonrib, or pelvic vs. nonpelvic primary disease. Extraosseous tumors did represent an unexpectedly high percentage of our cohort (22%), perhaps reflecting the wide age range of ES patients included in the analysis.

Strengths of our analysis are that it reflects near uniform treatment with contemporary pediatric and adult chemotherapy protocols, robust imaging assessment both prior to and following initial systemic therapy, tumor necrosis determination by a high-volume sarcoma pathology service, and few patients lost to follow-up over the study period. Limitations include lack of advanced imaging such as PET for the majority of our cohort, variability of radiotherapy techniques employed over the study period, and the broad inclusion criteria, perhaps reducing applicability to a specific age range. Further, a greater proportion of patients identified were treated from 2009 to 2019, suggesting that patients treated earlier in the study period may have been incompletely captured for analysis.

The importance of prompt initiation of neoadjuvant chemotherapy even in patients with radiographically localized Ewing sarcoma is due in part to the recognition that circulating tumor cells and radiographically undetectable sites of micrometastatic disease are often present at diagnosis [31]. The frequency of isolated pulmonary recurrences in our cohort and others suggests that micrometastases in the lungs may be an effective therapeutic target. Indeed, it has been demonstrated that patients with macroscopic pulmonary metastases at diagnosis have improved outcomes when they receive consolidative WLI [32, 33]. Further, other groups have identified the need to intensify therapy in patients with localized ES with high-risk prognostic features such as poor histological response (≥10% tumor viability) and size ≥200 mL, such as the implementation of high-dose chemotherapy with autologous stem-cell rescue performed under the Euro-E.W.I.N.G. and Ewing-2008 trials [7]. A prospective clinical trial of prophylactic WLI combined with modern multimodality therapy in patients at high risk for recurrence, as assessed by inferior radiographic or pathologic response to chemotherapy, could help to determine whether outcomes can be improved in these patients by therapeutic intensification targeting the lungs.

## Figures and Tables

**Table 1:** Patient, tumor, and treatment characteristics of localized Ewing sarcoma cohort.

		No relapse	Relapse
Number of patients		73	21

Age at diagnosis	Median age	15.8 years	21.1 years
	<10 y	19 (26%)	1 (5%)
	10–21 y	24 (33%)	9 (43%)
	>21 y	30 (41%)	11 (52%)

Primary tumor site	Appendicular	18 (25%)	6 (29%)
	Axial	38 (52%)	11 (52%)
	Extraosseous	17 (23%)	4 (19%)

Primary tumor size	Median size	6.4 cm	8.5 cm
	Mean size	7.1 cm	9.5 cm
	≤5 cm	25 (34%)	5 (24%)
	5.1–9.9 cm	31 (42%)	6 (29%)
	≥10 cm	14 (19%)	8 (38%)
	Unknown	3 (4%)	2 (10%)

Definitive treatment	Surgery alone	42 (58%)	9 (43%)
	RT alone	13 (18%)	5 (24%)
	Surgery + RT	18 (25%)	7 (33%)

Percent necrosis	≥95%	27 (37%)	2 (10%)
	<95%	14 (19%)	10 (48%)
	Unknown or N/A	32 (44%)	9 (43%)

**Table 2:** Patterns of failure and sites of first recurrence in patients with recurrence of initially localized or metastatic Ewing sarcoma.

		Localized	Metastatic
Number of patients		21	34

Metastatic site(s) at time of diagnosis	Lung only	—	11 (32%)
	Lung + other	—	10 (29%)
	Bone only	—	4 (12%)
	All other	—	9 (26%)

Pattern of first failure	Local	4 (19%)	1 (8%)
	Distant	14 (67%)	12 (54%)
	Both	3 (14%)	7 (38%)

Postrecurrence site(s) of distant metastases	Lung only	9 (53%)	4 (21%)
	Lung + other	5 (29%)	5 (26%)
	Bone only	2 (12%)	4 (21%)
	All other	1 (6%)	6 (32%)

## Data Availability

The datasets generated and/or analyzed during the current study are available from the corresponding author on reasonable request.
